# Novel Therapeutic Targets in Metabolic Disorders: From the Bench to the Bedside

**DOI:** 10.1155/2014/365974

**Published:** 2014-05-13

**Authors:** Maddalena Illario, Carolina Di Somma, Guido Iaccarino, Pietro Campiglia, Uma Sankar, Nunzia Montuori

**Affiliations:** ^1^Department of Translational Medical Sciences, University of Naples Federico II, 80131 Naples, Italy; ^2^IRCCS, SDN Foundation, Via Emanuele Gianturco 113, 80143 Napoli, Italy; ^3^Department of Medicine and Surgery, University of Salerno, 84084 Salerno, Italy; ^4^IRCCS “Multimedica”, Sesto San Giovanni, 20099 Milano, Italy; ^5^Department of Pharmaceutical Science, University of Salerno, Fisciano, 84084 Salerno, Italy; ^6^James Graham Brown Cancer Center and Owensboro Cancer Research Program, University of Louisville, Owensboro, KY 42303, USA; ^7^Department of Pharmacology and Toxicology, University of Louisville, Louisville, KY 40202, USA

The aim of this special issue is to explore innovative approaches to bridge laboratory investigation and clinical research in endocrine-metabolic disorders. “From bench to bedside” refers to the concept of “translational medical research”: within public health, translational medicine is indeed focused on ensuring that proven strategies for disease treatment and prevention are actually implemented within the community. Translational research also concerns how the process of transfer of innovations to daily use can be more effective and can speed up the impact of discoveries in the lives of patients. Another goal of this special issue was to encourage the multidisciplinarity efforts in health research: integration of evidence and knowledge from multiple sources and backgrounds enhances our understanding of health processes and carries the potential to generate breakthrough innovations to improve human health in the future.

It is an honor to invite you to enjoy reading this special issue.

One article was “*Possible potentiation by certain antioxidants of the anti-inflammatory effects of diclofenac in rats,*” where authors investigated the potential beneficial impact of the addition of antioxidant supplements to diclofenac regimen in a model of carrageenan-induced paw. In conclusion the combination of diclofenac and any of the anti-inflammatory agents tested appears to preserve the immunomodulating effect of the antioxidant alone. Thus, the authors conclude that the addition of antioxidants to any treatment regimen using this particular drug could have potential beneficial effects for the patients, albeit it does not build upon the effects from the diclofenac per se.

Another article was “*Detection of impaired cognitive function in rat with hepatosteatosis model and improving effect of GLP-1 analogs (exenatide) on cognitive function in hepatosteatosis.*” The aims of this study were to evaluate (1) detection of cognitive function changing in rat with hepatosteatosis model and (2) the effect of GLP-1 analog (exenatide) on cognitive function in hepatosteatosis. Authors concluded that memory performance falls off in rats with hepatosteatosis feeding with fructose (decreased latency time). However, GLP-1 ameliorated cognitive functions (increased latency time) in rats with hepatosteatosis and related metabolic syndrome. Indeed they also showed that exenatide treatment improved learning and memory performance in rats with hepatosteatosis and metabolic syndrome. So, this drug might be a candidate for alleviation of memory and cognitive dysfunctions in metabolic disorders.

In the article “*Evaluation of lung and bronchoalveolar lavage fluid oxidative stress indices for assessing the preventing effects of safranal on respiratory distress in diabetic rats*” authors investigated the effects of antioxidant activity of safranal, a constituent of* Crocus sativus* L., against lung oxidative damage in diabetic rats, concluding that safranal treatment may be effective to prevent lung damage in diabetic rats by modulation of oxidative stress. These findings support the efficacy of safranal as natural antioxidant for diabetes and its complication management.

The aim of the paper “*A novel role of globular adiponectin in treatment with HFD/STZ induced T2DM combined with NAFLD rats*” was to evaluate the effects of globular adiponectin (gAd) on treatment of type 2 diabetic rats combined with NAFLD. Authors concluded that globular adiponectin could ameliorate the hepatic steatosis and vary the expressions of adiponectin receptors in liver and skeletal muscle by stimulating insulin secretion.

In the review article “*Endocrinopathies after allogeneic and autologous transplantation of hematopoietic stem cells,*” authors summarized (1) main endocrine disorders reported in literature and observed in their center (Hematology and Hematopoietic Stem Cell Transplant Center in the Department of Medicine and Surgery of University of Salerno,) as consequence of auto- and allo-HSCT and (2) an outline of current options for their management. Their analysis further provide evidence that the main recognized risk factors for endocrine complications after HSCT are the underlying disease, previous pretransplant therapies, the age at HSCT, gender, total body irradiation, posttransplant derangement of immune system, and, in the allogeneic setting, the presence of graft-versus-host disease requiring prolonged steroid treatment.

Last but not least, in this special issue was also published the review article “*Targeting mitochondria as therapeutic strategy for metabolic disorders*” where authors analyzed the critical role of mitochondria as key regulators of cell metabolism.

